# Phylogenetic comparative and expression analysis of genes encoding dof transcription factors from *Eucalyptus grandis*

**DOI:** 10.1186/1753-6561-5-S7-P159

**Published:** 2011-09-13

**Authors:** Gabriel d'Almeida, Michèle Breton, Sandro Camargo, Jeverson Frazzon, Giancarlo Pasquali

**Affiliations:** 1 Biotechnology Center, Federal University of Rio Grande do Sul, Porto Alegre, RS, Brazil; 2 Institute of Informatics, Federal University of Rio Grande do Sul, Porto Alegre, RS, Brazil; 3 Institute of Food Science and Technology, Federal University of Rio Grande do Sul, Porto Alegre, RS, Brazil

## 

Dof proteins are a family of transcription factors specific to the plant kingdom that contain a particular class of zinc finger DNA binding domain. Members of this family are involved in the regulation of genes related to a plethora of metabolic processes including stress or hormone response, seed and endosperm development, flowering, carbohydrate metabolism, and cell or tissue specificity. Dof proteins and encoding genes were characterized in several plant species. Nevertheless poplar is the only woody species whose *Dof* genes were better characterized. The recent availability of the *Eucalyptus grandis* genome and transcriptome, along with transcription factor databases of several plant species allowed us to identify and run a valuable comparative analysis of the Dof protein family in this tree. These species included *Arabidopsis thaliana*, *Arabidopsis lyrata*, *Carica papaya*, *Populus trichocarpa*, *Vitis vinifera*, *Sorghum bicolor*, *Chlamydomonas reinhardtii*, *Oryza sativa indica* and *Zea mays*.The phylogenetic relationships among Dof proteins from *E. grandis* and *Arabidopsis thaliana* - a popular model for studying the genomics of many plants - is a fundamental step to unravel functionality of new *Dof* genes not yet characterized. Twenty-three distinct DNA sequences were predicted to belong to the *Dof* gene family after the analysis of the complete available genome of *E. grandis*. The deduced protein sequences of 22 members do contain a conserved Dof domain. One sequence seemed to have lost the conserved Dof domain, suggesting it to be a pseudogene or to present an activity not directly linked to the Dof family. Gene structures, including exon/intron positions, and amino acid sequences were predicted for each gene based on the available *E. grandis* transcriptome. In order to determine the relationship and function of the genes putatively encoding Dof proteins, we carried out a phylogenetic analysis with 43 Dof protein sequences from *A. thaliana*. Our analysis allowed us to classify the *E. grandis Dof* sequences into five groups of orthologous genes. Gene expression analysis via real time, quantitative PCR was also conducted with ten of the *E. grandis Dof* genes, using samples obtained from flowers, leaves and vascular tissue. Generally, *Dof* steady-state mRNA levels were higher in *E. grandis* vascular tissues, with more reduced levels in flowers. *Dof* genes showed an increase in steady-state mRNA levels after hormone signaling, and reduced levels following abiotic stress. This is the first study that aimed the identification of *Dof* genes in *E. grandis* which are possibly involved in numerous plant metabolic processes. The phylogenetic relationship to *A. thaliana* counterparts and the patterns of mRNA accumulation in *E. grandis* allowed us to speculate on possible roles for some of the Dof-encoding genes.

Financial support: Brazilian Ministry of Agriculture, Farming & Supply (MAPA), and The National Council for the Development of Science & Technology (CNPq), Brazilian Ministry of Science & Technology (MCT).

